# Examining Differential Effects of Digital Parent Training for Child Behavior Problems on Maternal and Paternal Outcomes

**DOI:** 10.3390/children12040469

**Published:** 2025-04-06

**Authors:** Or Brandes, Chen R. Saar, Amit Baumel

**Affiliations:** Department of Community Mental Health, University of Haifa, Haifa 3103301, Israel; orachm01@campus.haifa.ac.il (O.B.); csaar@campus.haifa.ac.il (C.R.S.)

**Keywords:** digital parent training, maternal and paternal outcomes, child behavior problems, parental differences

## Abstract

Background/Objectives: Parent training programs (PTPs) have long been recognized as effective interventions for early onset of child behavior problems, with evidence showing significant improvements in parenting practices and child outcomes. However, little is known about potential differences in treatment outcomes between mothers and fathers. This study examined changes in parenting practices and self-efficacy between mothers and fathers (*n* = 31 couples). Methods: Couples participated in a 10-week digital parent training program for child behavior problems. Both parents completed measures of parenting practices (Parenting Scale, Alabama Parenting Questionnaire) and self-efficacy (Parenting Tasks Checklist, Me as a Parent Scale) at baseline and post-intervention. Results: Significant, large-effect-size improvements in most parenting variables were found for both mothers and fathers (*p*_s_ ≤ 0.03, *η_p_*^2^ ≥ 0.15). No significant interaction effects were found between the parent (mother, father) and intervention time (pre, post-intervention) in parenting variables (*p* ≥ 0.18), indicating similar patterns of improvement. Mothers reported higher levels of positive parenting practices compared to fathers regardless of intervention time (*p* = 0.01, *η_p_*^2^ = 0.19). The initial severity of child behavior problems moderated differences between parents in improvements in sense of competence (*U* = 171.50, *p* = 0.03), with smaller mother–father gaps observed for families beginning with higher levels of child behavior problems. No other demographic variables moderated parent differences. Conclusions: Digital delivery format may help promote more equitable benefits for both parents. Further research with larger samples and longer follow-up time is needed to validate these preliminary findings.

## 1. Introduction

Parent training programs (PTPs) aimed at treating early onset of child behavior problems have shown efficacy in a large number of trials, leading to significant improvements in child behavior problems, parenting practices, and perceptions [1,2,3,4]. PTPs leans on the conceptual understanding that parenting perceptions, attitudes, and practices shape parent–child interactions and subsequently child behaviors [5,6]. For this reason, parenting styles are considered mechanisms of change in PTPs aimed at treating child behavior problems [7].

The literature often refers to parenting factors as ‘parental variables’, while predominantly studying maternal aspects without differentiation between mothers and fathers, likely because of the past emphasis on mothers as holding the main socializing and childcare roles. Systematic reviews and meta-analyses over the past few decades suggest that father involvement in parent training has remained low [8,9,10]. For instance, one systematic review found that 87% of identified studies of PTPs did not include information on father-related outcomes [8].

Most of the studies that did engage fathers in the intervention focused on examining how father participation can improve fathers’ parenting practices [10,11], maternal outcomes [12,13], the couple’s relationship [11,12], and child outcomes [10,12,14]. Yet few studies compared maternal and paternal outcomes directly [10,15]. Additionally, several reviews indicate that studies including fathers do not often report mother and father data separately [15,16,17,18].

Studies examining differences in parental outcomes following parent training revealed that the intervention may influence maternal and paternal behaviors differently. Lundahl et al. (2008) conducted a meta-analysis of parent training and reported that treatment gains were generally greater for mothers compared with fathers in terms of magnitude and the maintenance of effects [19]. Specifically, the mother’s parenting behaviors were found to improve significantly more than the father’s immediately following the intervention (Cohen’s ds = 0.84 and 0.53, respectively). This difference was also largely maintained at follow-up.

Subsequently, in a different yet related field of sleep-disturbed young children, a parent training program with active participation from both parents resulted in improvements in psychosocial health, coping strategies, and parental self-efficacy—predominantly among mothers. Fathers, by contrast, reported fewer improvements [20]. Similarly, another study found that mothers experienced significant improvements in their parental sense of competence and parent–child interaction, whereas fathers did not [21].

In summary, studies that included fathers indicate differential impacts of traditional PTPs on mothers and fathers, with mothers consistently reporting greater improvements in parenting practices, self-efficacy, and psychosocial outcomes. The differences in intervention outcomes between mothers and fathers may be attributed to how well the intervention aligns with the lifestyle and preferences of fathers. Given that PTPs were largely developed with mothers as the primary caregiver parent in mind [9], studies have highlighted the need to develop and implement PTPs that better address the specific needs and lifestyles of fathers to enhance their engagement and outcomes [9,22,23].

Subsequently, Panter-Brick et al. (2014) [10] suggested that issues related to intervention design (e.g., timing, place, and the medium of program delivery) should be considered by therapists to allow fathers’ active participation in parental interventions. Other studies have suggested that remotely delivered interventions, such as telehealth, offer greater flexibility and thus holds promise for father engagement [9,24]. Congruent with this notion, fathers tend to prefer less intensive delivery formats such as internet-based programs [22]. Digital parent training programs (DPTPs) can reduce barriers for fathers by providing flexible access through computers or smartphones, accommodating varied schedules, and minimizing travel requirements [25].

### The Current Study

To the best of our examination, studies in the field of digital parent training aimed at treating child behavior problems have not examined how these interventions specifically affect maternal and paternal outcomes when both parents actively participate. Addressing this gap is essential for developing more equitable and effective family-based interventions. The current study aims to close this gap in the literature by examining and comparing changes in both parents (mothers and fathers) separately following their participation in a digital parent training aimed at treating their child’s behavior problems. Additionally, the study aimed to examine whether demographic features will moderate intervention-induced differences between mothers and fathers in parenting related variables.

## 2. Materials and Methods

The current paper is a secondary analysis of a randomized controlled trial that was previously reported [26,27]. Parents were randomly allocated to one of two groups (1:1) through a computer-generated randomization process, with stratification based on child gender, by an independent researcher who was blinded to their assessments. Randomization and trial procedures were carried out in accordance with recommended guidelines (e.g., [28]; ClinicalTrial.gov registry number: NCT05344885), and approved by the institutional review board of University of Haifa (approval number: 058/22).

### 2.1. Recruitment Procedure

Recruitment took place from 1 May 2022, to 7 July 2022, via social media platforms and an sponsored advertising campaign on Facebook. Interested parents were referred to the project website for further information about the study. Parents who left their contact details on the website received a short eligibility screener with the exclusion criteria and eight items capturing their child’s behavior problems, based on the criteria for oppositional defiant disorder taken from the Diagnostic and Statistical Manual of Mental Disorders (DSM-V). Parents who marked concerns for at least 4 out of 8 behaviors considered as preliminarily eligible. Then, a research assistant confirmed screening, interest, and parents’ understanding of the terms of the study via a phone call. Eligible participants received access to the program web platform via personal login credentials. Then, participants were referred to a web-based informed consent form, which was attached to the following baseline assessment battery.

### 2.2. Participants

Parents were considered eligible to participate if they met the following criteria: (a) they had a child aged 3 to 7 years who exhibited (b) high levels of behavioral problems, as indicated by the Eyberg Child Behavior Inventory (ECBI) subscales (ECBI Problem ≥ 15 and ECBI Intensity ≥ 132 [29,30]); and (c) they owned a smartphone with cellular and Internet connectivity. Parents were excluded if they reported any of the following: (a) their child regularly received professional treatment or medication for behavioral or emotional issues; (b) they were currently receiving parenting support from another source; or (c) their child had been diagnosed with an intellectual disability or developmental delay. Parents who did not meet the eligibility criteria were referred to local services.

Parents from each participating family were encouraged to participate in the intervention in a joint active manner, with both spouses participating when feasible. In total, 37.5% (*n* = 33/88) of families in the original study decided to participate with both spouses. Families that chose to participate jointly were asked to choose a leading parent (i.e., the parent who spends most of the time with the child during the day and that will provide the username through which both parents enter the program). Of the 33 families that chose to participate with both parents, 31 families (93.93%) defined the mother as the ‘Leading Parent’. Since there was no statistical way to examine cases in which the father was defined as the leading parent, the current study analysis relied on the 31 families in which both parents participated and the mother was defined as the leading parent. All parental variables were measured separately both for mothers and fathers at baseline (pre-intervention) and at 10 weeks after baseline (post-intervention). Informed consent was obtained from all subjects involved in the study.

### 2.3. Intervention

**Figure 1:**
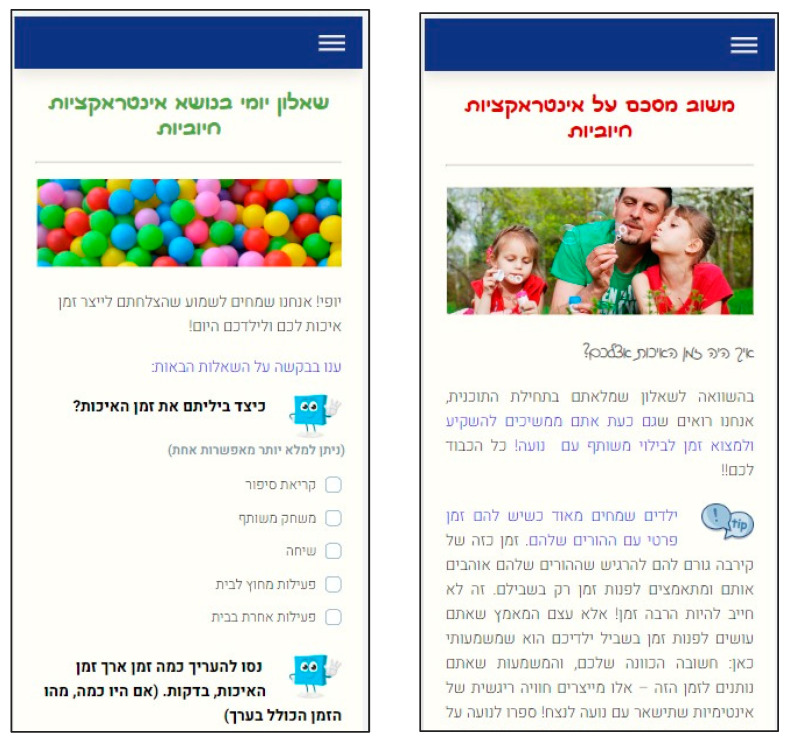
Mobile screenshot samples of a daily brief monitoring questionnaire (left screen) and personalized daily positive feedback (right screen; texts are in Hebrew).

Parents received one of two interventions that are comprehensively described in the primary outcomes paper and include the exact same content but differ in its delivery [26]. Because the current study involved comparison between parents who received the same intervention without any comparison between the two interventions, the following description will refer only to the content parents received during their participation. The content is based on a seven 10 to 25 min interactive e-learning modules, recommended to be completed within a ten-week period; each module discusses a specific theme: (1) introduction to parent training; (2) parent–child positive interactions and quality time; (3) parental emotion regulation; (4) building effective routines and clear ground rules; (5) recognizing positive behaviors/ignoring minor negative behaviors; (6) dealing with disobedience; and (7) the parent as a mentor and teacher. Recommendations for practicing the skills, “homework”, and additional features (i.e., downloadable materials) were presented at the end of each module. The interventions were delivered through MindTools, an open-source eHealth platform that was adapted and further upgraded by Prof. Amit Baumel and is available on GitHub [31]. Figure 1 presents a sample mobile screenshot of the program’s design and user interface.

### 2.4. Measures

The study instruments included self-reported questionnaires and automated documentation of program usage. All self-report measures were administered through ‘Qualtrics’. Demographic data was collected via baseline questionnaire. As mentioned above, the present report will refer to the data collected from families where both parents filled out separate questionnaires of parental variables.

Eyberg Child Behavior Inventory (ECBI) [32]: This was used to evaluate child behavior problems, utilizing the Intensity and Problem subscales of the 36-item inventory [29,30]. Caregivers rated the intensity of each behavior on a 7-point scale (1 = never to 7 = always) and whether the behavior was problematic (0 = no; 1 = yes). In this study, the internal consistency coefficients (alpha values) were as follows: ECBI Problem = 0.82, ECBI Intensity = 0.80.

The Parenting Scale (PS) [33]: This assessed parental disciplinary responses to child misbehavior through two subscales: Over-reactivity (11 items) and Laxness (10 items), which reflect ineffective discipline on either end of the spectrum. Parents rated items on a 7-point Likert scale. The internal consistency scores for this study were as follows: Laxness = 0.83, Over-reactivity = 0.75.

The Parenting Tasks Checklist (PTC) [21]: This measured task-specific self-efficacy using items from the setting self-efficacy (6 items) and behavioral self-efficacy (6 items) subscales. Responses were rated on a scale from 0 (Certain I can’t do it) to 100 (Certain I can do it). The internal consistency scores for this study were as follows: PTC Setting = 0.88, PTC Behavioral = 0.91.

Parental Self Efficacy (Me as a Parent; MaaP) [34]: This evaluated general self-efficacy using the 4-item Self-Efficacy subscale, with each item rated on a Likert scale from 1 (strongly disagree) to 5 (strongly agree). The internal consistency coefficient (alpha) for this subscale was 0.79 in the present study.

Alabama Parenting Questionnaire (APQ) Positive Parenting Practices [35]: This measured positive parenting practices using a 6-item subscale. Parents rated each item on a scale from 1 (never) to 5 (always) based on how frequently it occurs in their household. The internal consistency coefficient for this subscale was 0.93 in this study.

### 2.5. Statistical Analysis

Demographic and usage data are reported as frequencies and percentages for categorical variables and means and standard deviations for continuous variables. Differences in parental outcomes between fathers and mothers were calculated using a two-way ANOVA analysis, based on time (pre- and post-intervention) and parent (mother, father). Due to the relatively small sample size, moderation effects of differences between the two parents were tested using the Mann–Whitney U test, appropriate for detecting group differences without assuming normality [36]. Statistical analyses were carried out using SPSS version 26.0.

## 3. Results

Participant demographics are presented in Table 1. Among the participating families, child mean age at the beginning of the intervention was 4.9, and 61.3% of children were males. Mother mean age was 36.4. The mean number of modules parents completed was 5.43 (SD = 2.01). The average usage time of the program was 132.6 min (SD = 79.61), with an average number of 17.77 (SD = 13.14) logins, and 17.25 (SD = 12.58) unique logins.

Table 2 presents the analysis of variance effects based on measurement time (pre- and post-intervention) and parent (mother, father). Significant large-effect improvements were observed across most parental outcomes (*p* ≤ 0.03, *η_p_*^2^ ≥ 0.15). Positive parenting practices (APQ) did not significantly improve over time; however, mothers consistently reported higher levels than fathers, irrespective of the intervention phase (*p* = 0.01, *η_p_*^2^ = 0.19). There were no significant interaction effects between parent (mother vs. father) and time (pre and post) in the parenting variables (*p* ≥ 0.18). That is, mothers and fathers tended to improve in a similar manner following the intervention.

Table 3 presents the moderation effects of child behavior problems (at baseline) and demographic variables on the differences in changes in parental variables between mothers and fathers following the intervention. Moderation effects were calculated using the Mann–Whitney U test. Levels of child behavioral problems (i.e., ECBI-Intensity) at baseline were found to be a significant moderator of differences in improvements between mothers and fathers in sense of parental competence (i.e., MAAP; *U* = 171.50, *p* = 0.03). Families that began the program with higher levels of child behavior problems exhibited a smaller gap in MAAP improvement between mothers and fathers at post-intervention compared to families that started the program with lower levels of child behavior problems (in which mothers reported less competence compared to fathers). No other moderating demographic variables were found beyond that.

## 4. Discussion

The present study aimed to evaluate the outcomes for both mothers and fathers following their joint participation in a digital parent training program designed to address child behavior problems. While much of the existing literature on digital parent training tends to categorize parenting factors as ‘parental variables’, it predominantly focuses on maternal aspects without distinguishing between the therapeutic outcomes for mothers and fathers. This study sought to address this gap in the literature. The intervention resulted in significant, large improvements for both mothers and fathers, with similar patterns observed across parenting practices, self-efficacy, and disciplinary behaviors. This finding stands in contrast to previous literature that typically showed greater gains for mothers compared to fathers in traditional parent training formats [19,20]. In the present study, the effect sizes in both mothers and fathers were large and similar to that seen in mothers in Lundahl et al.’s (2008) meta-analysis (i.e., *η_p_*^2^ > 0.15 in the present study and Cohen’s *d* = 0.84 in Lundahl’s study) [19].

The similar improvement patterns observed in our study among mothers and fathers may be attributed to several unique aspects of the digital delivery format. First, digital interventions offer flexible engagement with program content, allowing parents to access materials at convenient times that accommodate varied work schedules and commitments. This flexibility may particularly benefit fathers, who commonly report work commitments and practical barriers (i.e., availability of childcare, transportation difficulties) as major barriers to their participation in conventional PTPs [37,38]. Additionally, fathers typically prefer less intensive formats, such as internet-based parenting programs [22,38]. Digital delivery might also help overcome barriers such as fathers’ attitudes and beliefs about help-seeking, for example, discomfort in seeking or receiving parenting assistance and concerns about judgment [38]. Although mothers and fathers showed similar patterns in parenting outcomes, differences in their levels or styles of engagement with the intervention might still exist. Future research should specifically measure these engagement behaviors to provide a deeper understanding of potential variations between parents and their relation to therapeutic outcomes.

Interestingly, while both parents showed similar patterns of improvement, mothers consistently reported higher levels of positive parenting practices (APQ) than fathers, regardless of the measurement time-point (pre- or post-intervention). This finding aligns with other studies indicating that mothers are more actively involved in parenting and employ more positive techniques [39]. Societal expectations and traditional gender roles may influence mothers’ tendency to perceive and report their parenting as more positive. As primary caregivers, mothers are often expected to engage in nurturing and caregiving activities, which are typically associated with positive parenting practices [39].

The moderator analysis revealed that demographic variables, including child gender, family size, religiosity, socioeconomic status, and working hours, did not moderate the differential effects between mothers and fathers. This finding implies that the digital intervention’s ability to benefit both parents equally extends across diverse family contexts and circumstances.

One exception was the baseline severity of child behavior problems (ECBI-Intensity), which was significantly associated with the different parental changes reported at post-intervention among mothers and fathers in parental sense of competence. Specifically, families that began the program with lower levels of child behavior problems were characterized by a higher disparity between the mother and father regarding sense of parental competence improvement (mother gained more competence compared to father) compared to families that started the program with higher levels of child behavior problems. This finding suggests that the severity of child behavior problems may create some challenges for achieving equivalent intervention benefits among both parents.

### Limitations

Several limitations should be considered when interpreting these findings. First, the sample size was small, particularly for the moderation analyses, limiting our ability to detect subtle effects or to generalize findings broadly. However, all non-significant results were obtained with *p*-values that were not marginal, which reduces the likelihood that significant results would become significant with a larger sample size. Second, the short follow-up period (10 weeks) does not allow us to determine the sustainability of observed improvements or potential divergence in long-term outcomes between mothers and fathers. Previous research by Lundahl et al. [19] indicated that treatment gains for fathers tended to diminish more rapidly than for mothers over time, raising the question of whether the similar short-term improvements we observed would be maintained equally for both parents. Future research should employ larger samples and incorporate extended follow-up assessments to validate the long-term effectiveness and sustainability of DPTPs for mothers and fathers. In the current study, all the families that participated chose to define mothers as the ‘leading parent’ of the intervention. It would be beneficial to investigate in future research whether there are any variations in outcomes when the father is defined as the ‘leading parent’.

Additionally, while both mothers and fathers exhibited similar patterns in parental variables, differences in their engagement levels or interaction styles with the program may still exist. In the current study, data on engagement were not collected separately for mothers and fathers. Future research should specifically monitor these engagement behaviors to offer a more comprehensive understanding of potential differences between parents.

Furthermore, our study did not fully investigate all theoretical dimensions that may enhance understanding of the mechanisms underlying differential parental outcomes and potential intervening variables. For example, fathers and mothers may adopt different approaches to parenting, particularly in how they communicate mistakes to their children and express emotions through external signals [40,41]. Specifically, the ways in which parents discuss their mistakes or demonstrate humility when addressing children’s behavioral issues may help explain variations between mothers and father’s outcomes.

## 5. Conclusions

This study provides evidence that DPTPs can effectively improve parenting practices for both mothers and fathers, with similar patterns of change. These findings do not fully align with previous research showing differential effects between mothers and fathers in traditional formats and suggest that digital delivery may offer unique advantages for promoting equitable parental engagement and outcomes. Given that this is a preliminary finding, additional research on the outcomes of digital interventions for mothers and fathers is necessary to validate these results. Examining changes in parenting variables among both parents separately is critical for understanding parent-specific responses.

Our findings have several practical implications for policymakers and program designers. DPTPs should be considered a viable alternative to traditional face-to-face formats, particularly when aiming to engage both parents equally. However, offering a digital format may not be sufficient to ensure active participation and equitable outcomes for fathers. Digital programs may benefit from tailoring support to the specific needs, preferences, and schedules of individual parents, rather than adopting a ‘one-size-fits-all’ family-level approach. With current technological capabilities, programs can offer personalized content, as well as individualized prompts and reminders, to address the needs of both mothers and fathers. This approach can help foster greater engagement from parents who might not typically take on the primary caregiving role.

## Figures and Tables

**Table 1:** Participant demographic characteristics at baseline.

**Categorical Variables**		***n* = 31 (%)**
Child gender	Male	19 (61.3%)
	Female	12 (38.7%)
House level income ^1^	≤18,000	18 (58.1%)
	>18,000	13 (41.9%)
Religiosity	Not Religious	18 (58.1%)
	Religious	13 (41.9%)
Weekly working/studying hours ^2^	≤40	14 (45.2%)
	>40	17 (54.8%)
**Continuous Variables**		**M (SD)**
Parent age (years) ^2^		36.4 (3.84)
Child age (years)		4.9 (1.28)
Number of children in family		2.68 (0.87)
ECBI Intensity at baseline		143.74 (23.70)

^1^ In Israeli shekels; ^2^ Refers to mothers.

**Table 2:** Analysis of Variance of child behavior problems outcome measures based on pre- and post-intervention and parent identity.

		Mother		Father		Time	*p*	*η_p_^2^*	Parent	*p*	*η_p_^2^*	Interaction	*p*
		(*n* = 31)		(*n* = 31)		F(1,30)			F(1,30)			F(1,30)	
		Pre	Post	Pre	Post								
PS Over	*M*	3.47	3.03	3.94	3.21	24.08	**<0.001**	**0.44**	2.60	0.12	0.08	1.87	0.18
	(*SD*)	(0.80)	(0.79)	(1.01)	(0.86)								
PS Laxness	*M*	3.10	2.68	3.30	3.05	5.33	**0.03**	**0.15**	1.61	0.21	0.05	0.34	0.57
	(*SD*)	(0.95)	(0.89)	(1.00)	(1.41)								
PTC Setting	*M*	68.56	75.62	67.01	75.44	7.56	**0.01**	**0.20**	0.07	0.79	0.00	0.13	0.72
	(*SD*)	(19.25)	(17.10)	(20.89)	(19.38)								
PTC Behavioral	*M*	54.56	67.76	57.66	70.79	19.76	**<0.001**	**0.40**	0.65	0.42	0.02	0.00	0.98
	(*SD*)	(22.08)	(21.91)	(21.19)	(21.09)								
MaaP	*M*	15.12	15.93	14.42	16.16	15.58	**<0.001**	**0.34**	0.25	0.62	0.00	1.82	0.19
	(*SD*)	(2.37)	(2.05)	(2.63)	(2.64)								
APQ	*M*	13.29	13.74	12.61	12.80	1.24	0.27	0.04	6.80	**0.01**	**0.19**	0.31	0.58
	(*SD*)	(1.75)	(1.48)	(2.09)	(2.32)								

Note. Significant results are in bold. Abbreviations. SD—standard deviation; PS Over—Parenting Scale Over-reactivity; PS Laxness—Parenting Scale Laxness; PTC Setting—Parenting Task Checklist Setting self-efficacy; PTC Behavioral—Parenting Task Checklist Behavioral self-efficacy; MaaP—Me as a Parent; APQ—Alabama Parenting Questionnaire.

**Table 3:** Moderation effects of differences between mothers and fathers in parental variables following the intervention.

	PS-Over		PS-Laxness		PTC Setting		PTC Behavioral		MaaP		APQ	
	Med	*U* ^1^ (*p*)	Med	*U* (*p*)	Med	*U* (*p*)	Med	*U* (*p*)	Med	*U* (*p*)	Med	*U* (*p*)
ECBI-Intensity Pre												
Under Med (*n* = 18)	−0.40	133.50 (0.51)	0.04	159.00 (0.10)	1.75	120.50 (0.89)	−2.75	130.00 (0.62)	−3.00	171.50 **(0.03)**	0	138.50 (0.39)
Above Med (*n* = 13)	0.30		0.45		−0.33		−0.50		1.00		0	
Child Gender												
Male (*n* = 19)	−0.40	127.50 (0.59)	0.09	110.00 (0.89)	5.00	79.00 (0.16)	−0.50	99.50 (0.56)	−1.00	107.50 (0.79)	0.00	102.00 (0.65)
Female (*n* = 12)	0.10		0.13		−3.83		−1.00		−0.50		−0.50	
No. of Children in Fam												
Under 2 (*n* = 15)	−0.40	141.50 (0.40)	0.72	76.50 (0.09)	−7.17	155.50 (0.16)	−2.67	145.00 (0.34)	0	126.00 (0.83)	0	162.50 (0.09)
2 and Above (*n* = 16)	−0.30		0.04		4.08		8.83		−1.00		0.50	
Level of Religiosity												
Not Religious (*n* = 18)	−0.50	142.00 (0.33)	0.13	102.50 (0.57)	−4.16	147.50 (0.23)	−3.00	162.00 (0.07)	−1.50	133.00 (0.54)	−1.00	158.00 (0.11)
Religious (*n* = 13)	0.30		0.09		3.17		9.83		0.00		0.00	
SES												
Low (*n* = 18)	−0.40	144.50 (0.27)	0.09	138.50 (0.34)	2.50	81.50 (0.15)	2.33	91.00 (0.31)	−0.50	107.00 (0.71)	0	136.00 (0.46)
High (*n* = 13)	−0.20		0.36		−2.00		−1.50		−1.00		0	
Weekly working hours ^2^												
Under 40 (*n* = 14)	−0.50	154.00 (0.17)	0.09	131.00 (0.65)	3.41	95.00 (0.36)	6.80	89.00 (0.25)	−0.50	126.00 (0.78)	0	126.50 (0.77)
Above 40 (*n* = 17)	0.10		0.18		−1.17		−2.67		−1.00		0	

Note. Significant results are in bold. Abbreviations. PS-Over—Parenting Scale Over-reactivity; PS-Laxness—Parenting Scale Laxness; PTC Setting—Parenting Task Checklist Setting self-efficacy; PTC Behavioral—Parenting Task Checklist Behavioral self-efficacy; MaaP—Me as a Parent; APQ—Alabama Parenting Questionnaire; ^1^ standardized value of the Mann–Whitney U test. ^2^ mother.

## Data Availability

The data are not publicly available due to privacy reasons. All the anonymized data can be made available at the specific request to the corresponding author at e-mail abaumel@univ.haifa.ac.il.

## Abbreviations

The following abbreviations are used in this manuscript:

PTPs	Parent training programs
DPTPs	Digital Parent training programs
PS	Parenting Scale
PTC	Parenting Task Checklist
MaaP	Me as a Parent
APQ	Alabama Parenting Questionnaire
ECBI	Eyberg Child Behavior Inventory
